# Why should sarcopenic obesity be included in a routine assessment during weight-management programmes?

**DOI:** 10.3389/fendo.2022.962895

**Published:** 2022-07-22

**Authors:** Marwan El Ghoch, Massimo Pellegrini

**Affiliations:** ^1^ Department of Nutrition and Dietetics, Faculty of Health Sciences, Beirut Arab University, Beirut, Lebanon; ^2^ Department of Biomedical, Metabolic and Neural Sciences, University of Modena and Reggio Emilia, Modena, Italy

**Keywords:** obesity treatment, sarcopenic obesity, resting energy expenditure, physical fitness, physical activity, weight-loss maintenance

## Introduction

Sarcopenic obesity (SO) is not a new term, as it was introduced in the early years of the current century (1). It is a condition that represents the co-existence of an increase in abnormal body fat (BF) deposition (i.e. obesity) (2) and a reduction in muscle mass and strength (3). Since then, SO has been widely investigated, particularly in terms of its association with weight-related comorbidities (4, 5), such as cardiovascular diseases (i.e., hypertension) (6), as well as type 2 diabetes (7) and dyslipidaemia (5). In addition to this, interest has recently grown in finding a suitable and adequate definition, as well as the diagnostic criteria for SO (8). With this aim, great effort has been made by the relevant scientific bodies that deal with obesity, as the European Society for Clinical Nutrition and Metabolism (ESPEN) and the European Association for the Study of Obesity (EASO) have developed a new consensus (9). Despite the fact that this report is not a conclusive one, it can however be considered a good starting point and a step forward in the direction of definition and diagnosis (9).

Over the last few years, several studies on SO among non-older adults in weight-management settings have been conducted, and it seems that the prevalence of this phenotype in patients with obesity who are seeking weight-loss treatment is relatively high, ranging between 30-50% (7). However, the impact of SO on weight-management clinical outcomes seems to be ignored (10).

In fact, it is still a matter of debate whether SO may form an obstacle to weight-management programmes. In other words, it remains unclear if patients with SO may have worse outcomes in terms of obesity treatment outcomes, i.e., attrition or dropout, weight loss and weight-loss maintenance, in comparison to those with obesity with the same body mass (i.e., body weight and body mass index), but not sarcopenia. With this aim, in the last five years, a large amount of well-designed research has been conducted on this topic.

**Figure 1:**
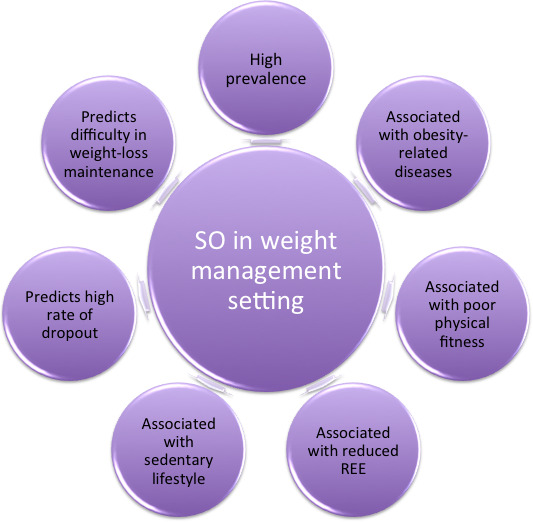
Rational behind the inclusion of SO in routine assessment in weight-management settings.

Based on these considerations, this opinion paper proposes to summarize the available literature on SO within weight-management settings, focusing on the impact of SO on variables involved in weight management, as well as clinical outcomes (**Figure 1**).

## Subsections relevant to the subject

### The association between SO and the main baseline weight-loss determinants: Physical fitness, resting energy expenditure and active lifestyle

For the success of any weight-management programme in terms of weight loss, an energy deficit should be created between caloric input and output (11); in other words, an increase in energy expenditure (physical activity and resting energy expenditure) (12) versus a decreased energy intake (dietary restriction) (12).

Physical fitness is defined as the ability to execute daily activities, and it is composed of five main components: cardiorespiratory fitness (CRF), muscular strength, muscular endurance, body composition and flexibility (13). For instance, a higher baseline CRF is associated with major weight loss in patients with severe obesity during an intensive lifestyle intervention programme (14). As such, physical fitness should be considered one of the main determinants of weight loss. A recent report showed that individuals with SO are characterized by reduced physical fitness, when compared to individuals with obesity but not sarcopenia (15).

The resting energy expenditure (REE) is defined as the required energy needed to maintain basic metabolic function (11). Since it represents nearly 70% of the total energy expenditure, the REE is considered one of the most important determinants of weight loss (11). A recent study showed that patients with SO displayed a significantly lower REE per unit body weight, by 1.5 kcal/day for each kg of body weight, than those in the group without SO (16).

Finally, strong evidence has shown the important role of regular physical activity (PA) (i.e., an active lifestyle) in long-term weight-loss maintenance. In fact, the only way to maintain weight loss in the long term is to be engaged in regular physical activity (17). In the same direction, SO seems to be associated with a lower number of routine daily steps and a sedentary lifestyle, when compared to individuals with obesity but not sarcopenia (18).

The findings on the negative association between SO and main baseline weight-loss determinants – namely impaired physical fitness and reduced resting energy expenditure, in addition to a sedentary lifestyle – opened up new directions for research focused on SO and weight-management programme outcomes, with the hypothesis that the latter may lead to poor weight-management outcomes.

### The relationship between SO and clinical outcomes: Attrition, weight loss and weight-loss maintenance

The two main outcomes in any obesity treatment during weight-management programmes are:

- Early attrition or dropout: This is considered one of the major causes of the failure of weight-management programmes for obesity. In some weight-loss programmes, this reaches nearly 80% (19).
- Long-term weight-loss maintenance (> 12 months): Nearly 70% of patients usually fail to maintain the amount lost during the weight-loss phase, and return to their baseline body weight after nearly three years of follow-up, regardless of the nature of the weight-loss treatment (20).

Hence it was worth examining whether patients seeking obesity treatment, and who are found to be affected by SO, are at a higher risk of interrupting the programme at an earlier stage or may have more difficulties in losing weight or maintaining the amount lost (21).

In fact, it seems that during an outpatient lifestyle modification weight-management programme for obesity, the presence of SO at baseline increases the risk of the early interruption of treatment (i.e., the risk of an early dropout at around six months) (22). This finding derives from a six-month longitudinal study that assessed the relationship between SO and early attrition in an outpatient weight-management programme for the treatment of obesity (22). The sample was composed of 103 adult patients with obesity (pooled from a large cohort). Of those, 72 interrupted the treatment during the weight-loss phase (before six months) and were considered to be “cases” in the “dropout group”. In addition, 31 patients (with a ratio of 2:1) with a similar body mass index (BMI) and the same gender, who had successfully completed the weight loss, were also selected from the same cohort to form a comparison group, and were regarded as “controls” in the “completer group” (22). The median age of the total sample was 35 (interquartile range (IQR) = 26.44) years, and nearly 70% were females. The authors found that the “dropout group”, when compared to the “completer group”, displayed a higher prevalence of SO (51.0% vs 25.8%). This was confirmed through a regression analysis that showed that SO increased the relative risk of dropout by nearly 150% after adjusting for age, gender, baseline BMI, and weight-loss expectation, as well as sedentary habits, in addition to the age at which they first began dieting (22).

Moreover, a study conducted by that research group in the same clinical setting assessed the relationship between SO and weight-loss outcomes, which was expressed as the percentage of weight loss (WL%), at six months as well as at a follow-up more than 12 months later, in 46 adult patients with obesity and a mean age of 44.25 ± 15.85 years composed mainly of females (78%) (23). At the six-month follow-up, participants with SO did not display a significant difference in terms of WL%, when compared to those without SO. However, after a longer term (i.e., >12 months), the WL% appeared to be significantly lower in the former group (SO vs non-SO) (−7.34 ± 6.29% vs −11.43 ± 4.31%; p = 0.024) (23). In fact, partial correlation analysis revealed a relationship between SO at the baseline and a lower WL% after more than 12 months (ρ = −0.425, p = 0.009), after controlling for age, sex, and BMI (23).

Therefore SO seems to be a factor that may affect the ability of patients with obesity to maintain their weight loss in the long term (i.e., after more than 12 months) in an outpatient weight-management clinical setting (23).

However, due to the small sample, these results are preliminary and need further replication in a larger population so that firm conclusions can be drawn. If confirmed, these findings may have relevant clinical implications for targeting patients with SO, who are more likely to drop out of treatment early on and/or face difficulties in maintaining the weight loss achieved during obesity treatment in the later stages. Therefore, implementing additional strategies for this subgroup of patients may be useful in order to obtain better clinical outcomes.

## Discussion

Despite the fact that the term SO was introduced into scientific literature more than two decades ago, it has not been sufficiently considered in “real-world” clinical settings and its assessment has been relatively ignored, especially in non-older adults with obesity. However, this phenotype was revealed to be prevalent in this particular population and setting (30-50%). In addition, there is a strong association between SO and weight-related diseases and comorbidities (i.e., type 2 diabetes, hypertension, dyslipidaemia) which should be managed promptly.

Moreover, in light of the recent literature published in the last five years – which certainly still needs further replication – it seems that SO is associated with a disadvantaged baseline profile (i.e., impaired physical fitness and reduced energy expenditure, as well as more sedentary behaviours), that may negatively impact the clinical outcomes, such as resulting in a higher rate of dropout, as well as major difficulties in long-term weight-loss maintenance.

To underline that point, higher adiposity, reduced lean mass and muscle strength are associated with poorer physical fitness (24). Moreover, it is known that skeletal muscle mass is one of the major determinants of REE (25). In addition, lower levels of PA are associated with higher BF and lower muscle mass (26). Since the SO phenotype is characterized by a combination of increased BF deposition, as well as a decrease in muscle mass and strength, this may explain why SO is associated with poor physical fitness, in addition to decreased REE, as well as a more sedentary lifestyle.

On the other hand, the reason behind the early dropout rate and lack of weight-loss maintenance in patients with SO is still unclear and needs further investigation. However, we can speculate that the above-mentioned disadvantaged baseline profile (impaired physical fitness and reduced REE as well as a sedentary lifestyle) may lead to (i) initial attenuated weight-loss rates that may not meet the patients’ weight-loss expectations, which is known to be a factor which can predict dropout (27); OR (ii) at a later stage, more difficulties in weight maintenance, especially due to the reduced level of physical activity, which is widely accepted as having a determinant role in long-term weight-loss maintenance (28).

The main findings reported in this current opinion paper should be interpreted with caution due to certain limitations: firstly, the data reported were obtained mainly from one outpatient weight-management unit based on a lifestyle modification programme, which means that external validation is required in different ones with similar settings, as well as in other clinical settings (i.e., inpatient) and treatment modalities (i.e., pharmacotherapy, bariatric surgery). Furthermore, these results, especially those related to a disadvantaged baseline profile as well as clinical outcomes, derive from relatively young adult patients with SO aged between 35-45 years and may not apply to older ones, especially if SO seems to be a more favourable condition when compared to sarcopenia alone in this population (i.e., older adults), for example, in terms of daily routine activities (29). In the same direction, the potential protective role of a higher body mass status (i.e., obesity) in older adults is still arguable in terms of important clinical outcomes (i.e., mortality, functional status, etc.) (30, 31). Therefore, weight loss in patients with SO remains a dilemma, especially for frail groups or older adults.

If these findings are confirmed in future studies, the screening/diagnosis of SO at the baseline of any weight-management programme should be considered vital, and including it in routine assessment during the evaluation of patients with obesity who are seeking weight-loss interventions will become crucial. Certain clinical implications stem from this observation. Firstly, awareness of this problem should be raised across heath care professionals dealing with obesity (i.e., clinicians, dieticians, nutritionists, etc.), as well as patients with the condition. Secondly, patients should be placed on personalized weight-loss programmes with specific strategies to overcome these obstacles, especially in the early stages, to prevent dropout, as well as in later phases of weight-management treatment to enhance weight-loss maintenance in the long term.

## Author contributions

All authors listed have made a substantial, direct, and intellectual contribution to the work and approved it for publication.

## Conflict of interest

The authors declare that the research was conducted in the absence of any commercial or financial relationships that could be construed as a potential conflict of interest.

## Publisher’s note

All claims expressed in this article are solely those of the authors and do not necessarily represent those of their affiliated organizations, or those of the publisher, the editors and the reviewers. Any product that may be evaluated in this article, or claim that may be made by its manufacturer, is not guaranteed or endorsed by the publisher.
